# Intra- and peritumoral radiomics features based on multicenter automatic breast volume scanner for noninvasive and preoperative prediction of HER2 status in breast cancer: a model ensemble research

**DOI:** 10.1038/s41598-024-55838-4

**Published:** 2024-02-29

**Authors:** Hui Wang, Wei Chen, Shanshan Jiang, Ting Li, Fei Chen, Junqiang Lei, Ruixia Li, Lili Xi, Shunlin Guo

**Affiliations:** 1 Department of Ultrasound, The First Hospital of Lanzhou University, Lanzhou, Gansu China; 2 The First Clinical Medical College, Lanzhou University, Lanzhou, Gansu China; 3 Department of Ultrasound, The Ningxia Hui Autonomous Region People’s Hospital, Yinchuan, Ningxia China; 4 Department of Advanced Technical Support, Clinical and Technical Support, Philips Healthcare, Xi’an, Shanxi China; 5 Department of Radiology, The First Hospital of Lanzhou University, Lanzhou, Gansu China; 6 Department of Pharmacologic Bases, The First Hospital of Lanzhou University, Lanzhou, Gansu China

**Keywords:** Cancer, Computational biology and bioinformatics, Oncology

## Abstract

The aim to investigate the predictive efficacy of automatic breast volume scanner (ABVS), clinical and serological features alone or in combination at model level for predicting HER2 status. The model weighted combination method was developed to identify HER2 status compared with single data source model method and feature combination method. 271 patients with invasive breast cancer were included in the retrospective study, of which 174 patients in our center were randomized into the training and validation sets, and 97 patients in the external center were as the test set. Radiomics features extracted from the ABVS-based tumor, peritumoral 3 mm region, and peritumoral 5 mm region and clinical features were used to construct the four types of the optimal single data source models, Tumor, R3mm, R5mm, and Clinical model, respectively. Then, the model weighted combination and feature combination methods were performed to optimize the combination models. The proposed weighted combination models in predicting HER2 status achieved better performance both in validation set and test set. For the validation set, the single data source model, the feature combination model, and the weighted combination model achieved the highest area under the curve (AUC) of 0.803 (95% confidence interval [CI] 0.660–947), 0.739 (CI 0.556,0.921), and 0.826 (95% CI 0.689,0.962), respectively; with the sensitivity and specificity were 100%, 62.5%; 81.8%, 66.7%; 90.9%,75.0%; respectively. For the test set, the single data source model, the feature combination model, and the weighted combination model attained the best AUC of 0.695 (95% CI 0.583, 0.807), 0.668 (95% CI 0.555,0.782), and 0.700 (95% CI 0.590,0.811), respectively; with the sensitivity and specificity were 86.1%, 41.9%; 61.1%, 71.0%; 86.1%, 41.9%; respectively. The model weighted combination was a better method to construct a combination model. The optimized weighted combination models composed of ABVS-based intratumoral and peritumoral radiomics features and clinical features may be potential biomarkers for the noninvasive and preoperative prediction of HER2 status in breast cancer.

## Introduction

Globally, breast cancer is the malignant disease with the highest incidence in women^1^. Breast cancer is a highly heterogeneous disease, and individualized clinical decision-making is particularly vital. Human epidermal growth factor receptor 2 (Human epidermal growth factor receptor2, HER2) positive is a molecular subtype of breast cancer. Compared with other subtypes, HER2-positive breast cancer has stronger heterogeneity, poorer prognosis, and lower survival rate^2^. HER2 positivity has been confirmed to promote tumor neovascularization and lymphangiogenesis, thereby affecting tumor growth and metastasis^3^. Chemotherapy combined with trastuzumab and pertuzumab is currently a first-line targeted therapy program for HER2-positive patients and can significantly improve patient outcomes^4^. Therefore, HER2 positive can reflect the information on tumor growth and metastasis, and its accurate judgment is crucial for the treatment and prognosis of breast cancer.

Automatic breast volume scanner (ABVS) automatically obtained three-dimensional ultrasound images of the breast using a standardized procedure. ABVS can obtain continuous ultrasound images, which are not influenced by the sonographer's operating technique and clinical experience. ABVS was found to have a higher value than conventional ultrasound in breast cancer screening^5^, observer consistency^6^, and preoperative prediction of neoadjuvant chemotherapy response^7^. Radiomics can analyze the potential relationship between medical imaging and tumor phenotype through high-throughput extraction of a large number of medical imaging features^8^. Previous studies on molecular subtypes of breast cancer had focused on the internal features of breast tumors, ignoring the peritumoral information^9,10^. Peritumoral tissues contain higher levels of markers for transcription factor activators, angiogenesis, proliferation, and invasion than tumor tissues, which determine tumor recurrence^11,12^. Studies had also demonstrated that peritumoral features were associated with the tumor necrosis factor (TNF) signaling pathway, which was involved in tumor angiogenesis, invasion, and metastasis^13^. Thus, the peritumoral region may embed biological information about tumor growth, invasion, and metastasis and may be a potential biomarker. In addition, studies also confirmed differences in Magnetic resonance imaging (MRI) findings of intratumoral and peritumoral among different molecular subtypes of breast cancer^14^. Therefore, the utilization of intratumoral and peritumoral features to predict HER2 status should have strong theoretical feasibility.

Some recent studies that predicted HER2 status in breast cancer focused only on radiomics features and did not involve clinically relevant data, which might affect model performance^15,16^. Model ensemble was an important strategy to optimize models, but the method of model weighted combination belonged to the model ensemble category was less applied in the field of predicting of HER2 status in breast cancer. Therefore, the present study aimed to construct models to predict the HER2 status of breast cancer by utilizing model weighted combination and feature combination methods based on ABVS intratumoral and peritumoral imaging features and clinically relevant features, to obtain the optimal model and provide the best basis for clinical decision-making for breast cancer patients.

## Materials and methods

**Figure 1:**
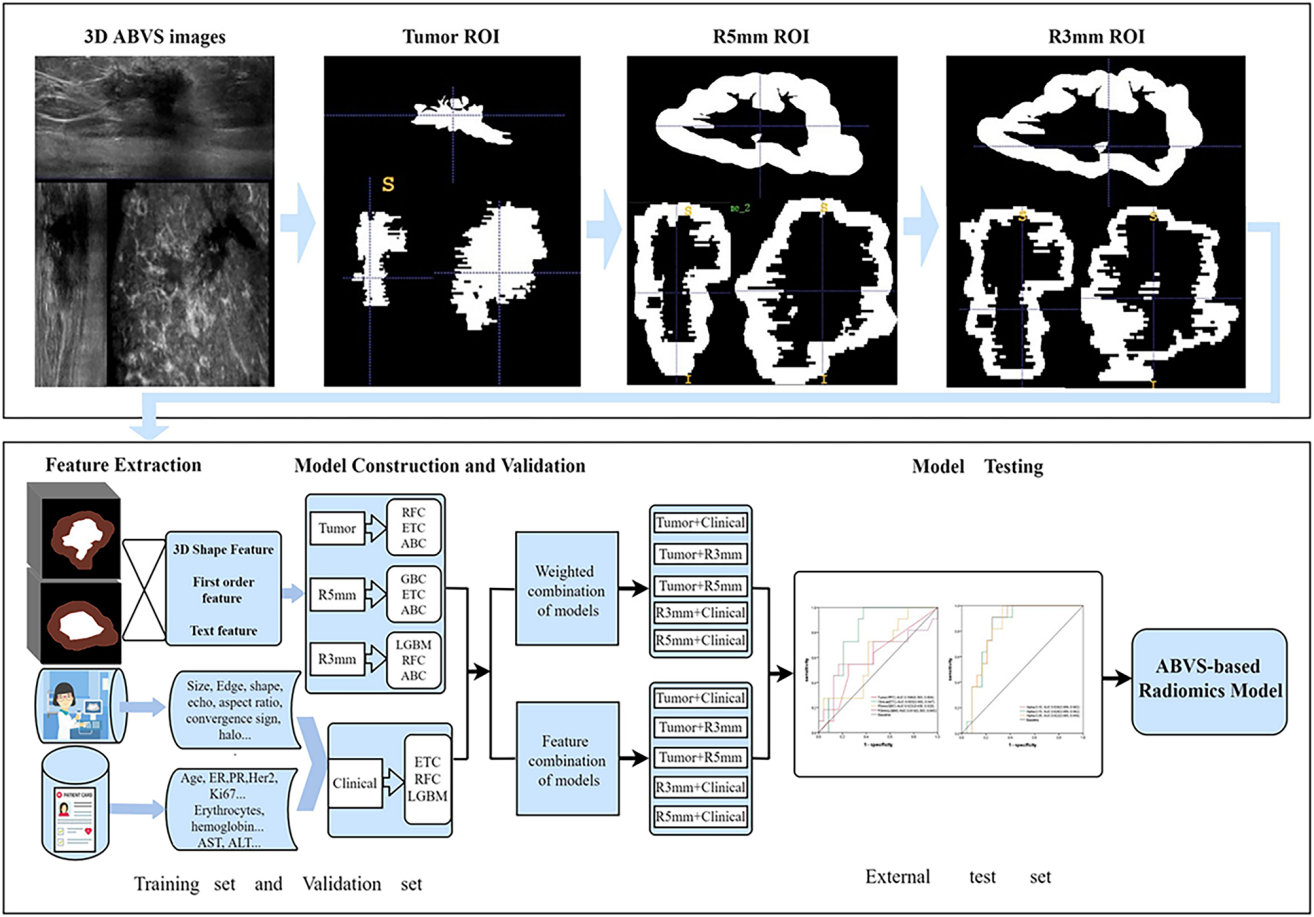
Overview of research protocol. Notes: R3mm, model based on peritumoral 3 mm ring of breast tumor; R5mm, model based on peritumoral 5 mm ring of breast tumor; Tumor, model based on radiomics features of the tumor; R5mm+Clinical, model based on radiomics features of the peritumoral 5 mm ring of breast tumor combined with clinical, ABVS, and serology features of breast tumor; R3mm+Clinical, model based on radiomics features of the peritumoral 3 mm ring of breast tumor combined with clinical, ABVS and serology features of breast tumor; Tumor+Clinical, model based on radiomics features of the tumor combined with clinical, ABVS and serology features of breast tumor; Tumor + R5mm, model based on radiomics features of the tumor and those of peritumoral 5 mm ring of breast tumor; Tumor+R3mm, model based on radiomics features of the tumor and those of peritumoral 3 mm ring of breast tumor. ABC, Ada Boosting Classifier; Clinical, Model based on clinical, ABVS, and serology features of breast tumor; ETC, Extra Tree Classifier; GBC, Gradient Boosting Classifier; LGBM, Light Gradient Boosting Machine; RFC, Random Forest Classifier.

The study was conducted in accordance with the Declaration of Helsinki and approved by the Review Board of the First Hospital of Lanzhou University. Because of the retrospective study, the Ethics Committee of the First Hospital of Lanzhou University exempted written informed consent. Figure 1 showed a flow chart of the research protocol.

### Patients

**Figure 2:**
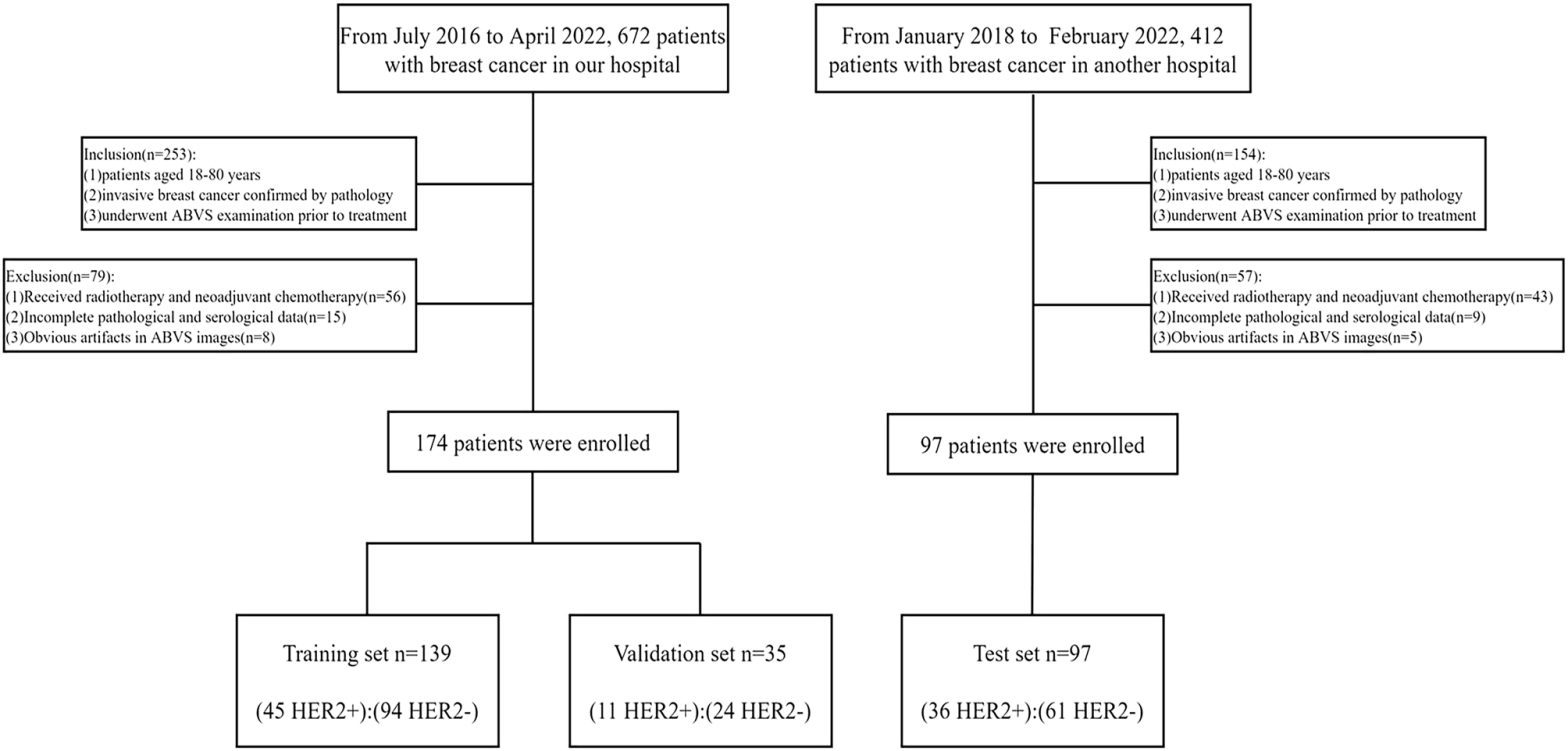
Flow chart of recruiting patients. HER2, Human epidermal growth factor receptor 2.

174 patients with invasive breast cancer confirmed in the First Hospital of Lanzhou University from July 1th, 2016 to April 30th, 2022 and 97 patients with invasive breast cancer confirmed in the Ningxia Hui Autonomous Region People's Hospital were collected in this study, which were conducted on May 1–5th, 2022. We had access to the information identifying each patient during or after data collection. Of these, 174 patients in our hospital were randomly divided into the training set and validation set (ratio 8:2), and 97 patients in the external hospital as the test set. Inclusion criteria were: (1) female patients aged between 18 and 80 years; (2) pathologically confirmed invasive breast cancer; (3) ABVS examination before treatment. Exclusion criteria were: (1) radiation therapy, neoadjuvant chemotherapy, or interventional therapy before ABVS examination (n = 48); (2) incomplete clinical, pathological, and serological information (n = 15); and (3) significant artifacts of the tumor area on ABVS images (n = 8). Finally, 174 patients with invasive breast cancer (all female, mean ± standard deviation: 48.8 ± 10.8 years) were included (Fig. 2, Table 1).

**Table 1:** Clinicopathological characteristics of patients.

Characteristics	Training set (N = 139)		*P* value	Validation set (N = 35)		*P* value
	HER2+ (n = 45)	HER2− (n = 94)		HER2+ (n = 11)	HER2− (n = 24)	
Age (years)	48.11 ± 8.88	48.88 ± 12.07	0.703^a^	50.00 ± 7.44	49.21 ± 10.28	0.821^a^
Postoperative axillary lymph node metastasis			0.017^a^*			0.357^a^
Positive	7	33		2	8	
Negative	38	61		9	16	
Histological grade			0.601^b^			0.323^b^
I	3	3		1	1	
II	24	55		3	13	
III	18	36		7	10	
ER			0.000^b^*			0.000^b^*
Positive	21	78		6	20	
Negative	24	16		5	4	
PR			0.000^b^*			0.149^b^
Positive	10	61		5	17	
Negative	35	33		6	7	
Ki-67			0.096^b^			0.552^b^
Positive (≥ 14%)	42	78		10	20	
Negative (< 14%)	3	16		1	4	

Significant values are in bold.

ALNM, axillary lymph node metastasis; ER, estrogen receptor; PR, progesterone receptor; HER2, human epidermal growth factor receptor 2.

^a^T-test.

^b^Chi-square test.

*P value < 0.05.

### Data acquisition

All patients underwent continuous cross-sectional scanning of each breast (interlayer spacing set at 0.5 mm) using a 14L5BV probe (7 MHz, dynamic range 50–55 dB) and a 14L5 linear probe (7–14 MHz frequency range and 10 MHz frequency center) of the ABVS (Siemens, AusonS2000, Munich, Bavaria, Germany), and the acquired axial plane images were transmitted to the workstation to automatically reconstruct sagittal plane and coronal plane images. All examinations were performed by a sonographer with 8 years of experience in ABVS examination. The results of immunohistochemistry (IHC) or fluorescence in situ hybridization (FISH) of breast cancer were considered the reference standard for HER2 status. The following clinical and serological indicators of patients were obtained from the electronic medical record system: age, erythrocytes, hemoglobin, hematocrit, mean erythrocyte volume, mean hemoglobin content, mean hemoglobin concentration, erythrocyte distribution width (standard deviation [SD]), erythrocyte distribution width (coefficient of variation [CV]), leukocytes, percentage of lymphocytes, percentage of monocytes, percentage of neutrophils, percentage of eosinophils, percentage of basophils, the absolute value of lymphocytes, the absolute value of monocytes, the absolute value of neutrophils, the absolute value of eosinophil, the absolute value of basophil, platelets, platelet ratio, mean platelet volume, platelet distribution width, large platelet ratio, cancer antigen 153(CA153), cancer antigen 125(CA125), carcinoembryonic antigen (CEA), total bilirubin, direct bilirubin, and indirect bilirubin.

### Region of interest (ROI) marking and ABVS ultrasonic feature extraction

Tumor ROIs were obtained through continuous manual 3D segmentation of breast tumors in the ABVS axial plane by two sonographers (with 8 years and 5 years of ABVS experience) using 3DSlicer version 4.11.2 (BWH, Boston, Massachusetts, USA), and then the ROIs of the 3 mm peritumoral and 5 mm peritumoral were acquired through the 3DSlicer editing function. Two sonographers assessed and recorded ABVS ultrasound features based on the Breast Imaging Reporting and Data System (Breast Imaging Reporting and Data System, BI-RADS), including tumor maximum diameter in the coronal plane, margin, shape, aspect ratio, halo, internal composition, echo, microcalcification, and convergence sign (coronal plane). For undetermined cases, the two sonographers reached a consensus through consultation. Two sonographers were unaware of the HER2 status of breast cancer.

### Radiomics feature extraction and model construction

The radiomics features of tumor ROI, 3mm peritumoral ROI, and 5mm peritumoral ROI were extracted using the Radiomics module of IntelliSpace Medicina Scientia (ISMS) version 2.4.0 (Philips Healthcare, Beijing, China) developed based on pyradiomics^17^. Image types included original, log, and wavelet images. Feature classes contained three-dimensional shape, neighborhood gray-tone difference matrix (Neighboring gray-tone difference matrix, NGTDM), gray dependence matrix (Gray-level dependence matrix, GLDM), gray level co-occurrence matrix (Gray-level co-occurrence matrix, GLCM), first sequence (First order), gray-level run-length matrix (Gray-level run-length matrix, GLRLM) and gray level area matrix (Gray-level size zone matrix, GLSZM). Given the relatively small proportion of HER2-positive cases (45/139) in the training set, the models were trained using the method of oversampling^18^. Through the Automatic Machine Learning (AML) function of ISMS version 2.4.0, models were constructed based on the radiomics features of the tumor, 3mm peritumoral region, 5mm peritumoral region, and the clinical features (clinical, ABVS ultrasound, and serological features), which were named as Tumor model, R3mm model, R5mm model, and Clinical model, respectively. Among the 13 classifiers of ISMS software, the best classifiers of four types of data sources (the highest sum of the AUC of the training set and the validation set of the classifiers) were selected for constructing four types of data source models.

### Radiomics model construction and optimization

The model weighted combinationand feature combination methods were used to construct and optimize the radiomics models. First, based on the four types of data source models, the weighted combination models of Tumor combined with Clinical (Tumor + Clinical), Tumor combined with R3mm (Tumor + R3mm), Tumor combined with R5mm (Tumor + R5mm), R3mm combined with Clinical (R3mm + Clinical), and R5mm combined with Clinical (R5mm + Clinical) were constructed and optimized using the method of a weighted combination of two data source models. In the validation set, alpha-AUC scatter plots of weighted models were plotted depending on the weighting coefficients (alpha), where two model results were combined using alpha*model 1 + (1-alpha) * model 2, to determine the optimal weighting coefficient and AUC of the weighted combination models.

Then, for the above four types of features, Tumor + Clinical, Tumor + R3mm, Tumor + R5mm, R 3 mm, R 5 mm + Clinical models were constructed and optimized using the feature combination method based on a variety of classifiers, and the performance of which was verified in the validation set.

### Radiomics model testing

In the test set, the weighted combination models and feature combination models were tested for predictive performance.

### Statistical methods

Statistical analyses were performed using SPSS version 24.0 (IBM, Armonk, NY, USA) and R software version 4.0.2 (MathSoft, Seattle, Washington, USA). For non-normally distributed variables, the Mann Whitney U test was utilized to compare statistical differences between the two groups. For normally-distributed variables, t-tests or chi-square tests were conducted. SPSS version 24.0 was performed to draw the Receiver operating characteristic curve (ROC). When the optimization function (0.6 * sensitivity + 0.4 * specificity) was maximum, the cutoff value, sensitivity, and specificity of weighted combination models were taken, and while the Yoden Index was maximum, those of the single data source model and feature combination model was acquired. Delong test was performed to compare the differences in AUCs between two models. R software version 4.0.2 was conducted to draw the scatter plots of the alpha-AUC.

## Results

### Baseline characteristics of patients

The baseline characteristics of the patients were listed in Table 1, S1 Table, and S2 Table, respectively. There were significant differences (*P* < 0.05) in shape, aspect ratio, mean erythrocyte volume, and mean hemoglobin content between HER2-positive and HER2-negative groups in the training and validation sets. In the training set, there were significant differences (*P* < 0.05) in margins, halos, microcalcifications, leukocytes, and the absolute value of neutrophils between the two groups. In the validation set, there were significant differences (*P* < 0.05) between the two groups in tumor maximum diameter in the coronal plane, hemoglobin, erythrocyte pressure, basophil percentage, platelet ratio, platelet distribution width, and large platelet ratio. In the training and validation sets, 45 cases (45/139) and 11 cases (11/35) patients were HER2-positive breast cancers, respectively.

### Comparison of the single data source models

**Figure 3:**
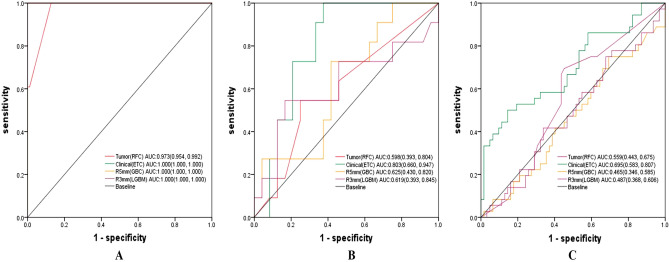
(**A**) ROCs of the optimal Tumor, the optimal R3mm, the optimal R5mm, and the optimal Clinical model in the training set. (**B**) ROCs of the optimal Tumor, the optimal R3mm, the optimal R5mm, and the optimal Clinical model in the validation set. (**C**) ROCs of the optimal Tumor, the optimal R3mm, the optimal R5mm, and the optimal Clinical model in the test set. R3mm, model based on peritumoral 3 mm ring of breast tumor; R5mm, model based on peritumoral 5 mm ring of breast tumor; Tumor, model based on radiomics features of the tumor. ABC, Ada Boosting Classifier; AUC, area under the curve; CI, confidence interval; Clinical, model based on clinical, ABVS, and serology features of breast tumor; ETC, Extra Tree Classifier; GBC, Gradient Boosting Classifier; LGBM, Light Gradient Boosting Machine; RFC, Random Forest Classifier.

The single-data source models were constructed respectively based on ISMS software. The ROCs of these models in the training set, validation set, and test set are shown in Fig. 3, Table 2. Thus, random forest classifier (RFC), light gradient booster (LGBM), gradient enhancement classifier (GBC), and Extra tree classifier (ETC) were the best classifiers to construct the Tumor model, R3mm model, R5mm model, and Clinical model, respectively. Overall, the AUCs of models decreased sequentially in the training, validation, and test sets. In the validation set, the Clinical model was the highest in terms of AUC 0.803 (95% confidence interval [CI] 0.660–0.947), with a sensitivity of 100% and specificity of 62.5%, followed by R5mm, R3mm, and Tumor models. In the test set, the Clinical model acquired the highest AUC of 0.695 (95% CI 0.583–0.807), the sensitivity of 86.1%, and specificity of 41.9%, followed by the Tumor, R3mm, and R5mm models.

**Table 2:** Predictive performance for HER2 state of single data source models based on a variety of classifiers, in the training, the validation, and the test set.

Single data source models	Classifiers	Data sets	AUC (95% CI)	Cutoff	Sensitivity	Specificity
Tumor	RFC	Training	**0.973 (0.954, 0.922)**	0.4966	1.000	0.832
		Validation	**0.598 (0.393, 0.804)**	0.4258	0.545	0.250
		Test	0.559 (0.443, 0.675)	0.3001	0.694	0.548
	ETC	Training	0.977 (0.973, 0.985)	0.4755	1.000	0.957
		Validation	0.555 (0.371, 0.739)	0.4055	1.000	0.292
		Test	0.516 (0.392.0.640)	0.3497	1.000	0.016
	ABC	Training	1.000 (1.000, 1.000)	0.5024	1.000	0.000
		Validation	0.549 (0.336, 0.763)	0.4841	0.636	0.625
		Test	0.531 (0.410, 0.652)	0.4265	0.944	0.177
Clinical	ETC	Training	**1.000 (1.000, 1.000)**	0.5996	1.000	0.000
		Validation	**0.803 (0.660, 0.947)**	0.3358	1.000	0.625
		Test	0.695 (0.583, 0.807)	0.2803	0.861	0.419
	RFC	Training	1.000 (1.000, 1.000)	0.5706	1.000	0.000
		Validation	0.754 (0.595, 0.913)	0.4457	0.909	0.667
		Test	0.705 (0.601, 0.809)	0.3522	0.889	0.468
	LGBM	Training	1.000 (1.000, 1.000)	0.5302	1.000	0.000
		Validation	0.667 (0.479, 0.855)	0.0608	0.909	0.417
		Test	0.722 (0.613, 0.831)	0.0373	0.972	0.258
R5mm	GBC	Training	**1.000 (1.000, 1.000)**	0.5003	1.000	0.000
		Validation	**0.625 (0.430, 0.820)**	0.0058	1.000	0.250
		Test	0.465 (0.346, 0.585)	0.000	1.000	0.000
	ETC	Training	1.000 (1.000, 1.000)	0.5667	1.000	0.000
		Validation	0.625 (0.409, 0.841)	0.1939	1.000	0.125
		Test	0.472 (0.352, 0.593)	0.0582	1.000	0.032
	ABC	Training	1.000 (1.000, 1.000)	0.5046	1.000	0.000
		Validation	0.606 (0.372, 0.840)	0.4815	0.727	0.500
		Test	0.472 (0.349, 0.596)	0.3547	1.000	0.016
R3mm	LGBM	Training	**1.000 (1.000, 1.000)**	0.5293	1.000	0.000
		Validation	**0.619 (0.393, 0.845)**	0.4159	0.545	0.833
		Test	0.487 (0.368, 0.606)	0.000	1.000	0.000
	RFC	Training	1.000 (1.000, 1.000)	0.5064	1.000	0.000
		Validation	0.614 (0.414, 0.813)	0.3730	1.000	0.292
		Test	0.494 (0.374, 0.614)	0.1290	1.000	0.048
	ABC	Training	0.998 (0.996, 1.000)	0.4945	1.000	0.968
		Validation	0.606 (0.422, 0.790)	0.4707	1.000	0.250
		Test	0.483 (0.368, 0.599)	0.4088	1.000	0.032

Bold characters represented the classifier with the highest sum of AUCs in the validation set and the test set.

R3mm, model based on peritumoral 3 mm ring of breast tumor; R5mm, model based on peritumoral 5 mm ring of breast tumor; Tumor, model based on radiomics features of the tumor; ABC, Ada Boosting Classifier; AUC, the area under the curve; CI, confidence interval; Clinical, model based on clinical, ABVS, and serology features of breast tumor; ETC, Extra Tree Classifier; GBC, Gradient Boosting Classifier; LGBM, Light Gradient Boosting Machine; RFC, Random Forest Classifier.

### Comparison of the weighted combination models

**Figure 4:**
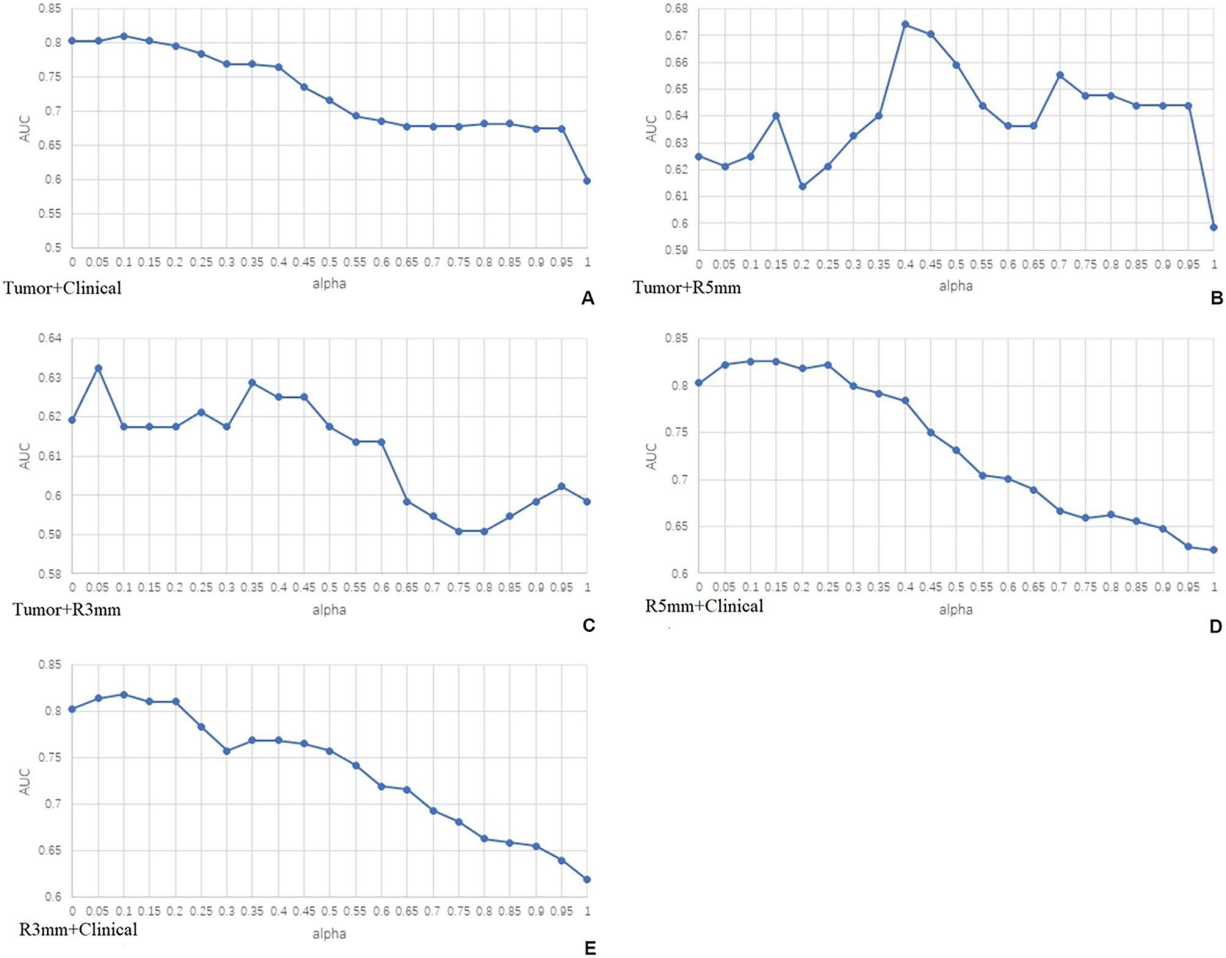
Scatter plots of alpha-AUC of weighted combination models in the validation set. (**A**) Scatter plots of alpha-AUC of the Tumor model combined with the Clinical model in the validation set. (**B**) Scatter plots of alpha-AUC of the Tumor model combined with the R5mm model in the validation set. (**C**) Scatter plots of alpha-AUC of the Tumor model combined with the R3mm model in the validation set. (**D**) Scatter plots of alpha-AUC of the R5mm model combined with the Clinical model in the validation set. (**E**) Scatter plots of alpha-AUC of the R3mm model combined with the Clinical model in the validation set. Notes: The two single data source models were combined through the formula of alpha*model 1 + (1-alpha) * model; Alpha, weighted coefficient. AUC, the area under the curve.

**Figure 5:**
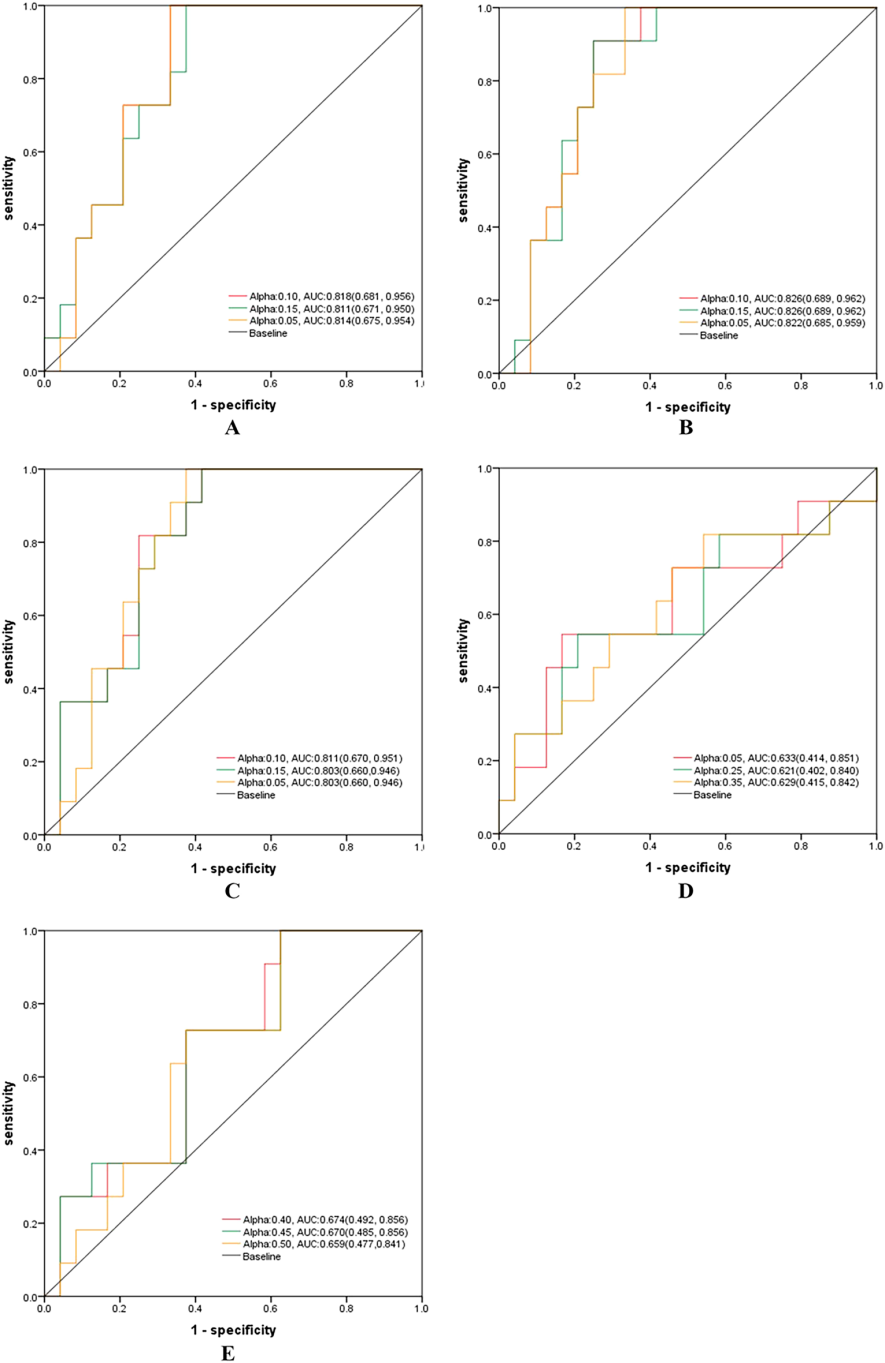
ROCs of weighted combination models based on different alphas in the validation set. (**A**) ROC of the R3mm model combined with the Clinical model in the validation set. (**B**) ROC of the R5mm model combined with the Clinical model in the validation set. (**C**) ROC of the Tumor model combined with the Clinical model in the validation set. (**D**) ROC of the Tumor model combined with R3mm model in the validation set. (**E**) ROC of the Tumor model combined with R5mm model in the validation set. Notes: Alpha, weighting coefficient. AUC, the area under the curve.

In the validation set, the model weighted combination method was adopted. It could be seen from the alpha-AUC scatter plots that Tumor + Clinical, R3mm + Clinical, and R5mm + Clinical achieved higher AUC when alpha was 0.10, 0.15, and 0.05; Tumor + R3mm owned higher AUC when alpha was 0.05, 0.25, and 0.35; Tumor + R5mm acquired better AUC when alpha was 0.40, 0.45 and 0.50 (Fig. 4, Table 3). In the validation set, the R5mm + Clinical model acquired the highest AUC, 0.826 (95% CI 0.689–0.962), the sensitivity of 100%, and specificity of 62.5%, which was sequentially higher than the R3mm + Clinical model, Tumor + Clinical model, Tumor + R5mm model, and Tumor + R3mm model (Fig. 5, Table 3).

**Table 3:** Predictive performance for HER2 state of weighted combination models based on different alphas, in the training, the validation, and the test set.

Weighted combination models	Sets	Alpha	AUC (95% CI)	Cutoff	Sensitivity	Specificity
R3mm + Clinical	Validation	0.10	0.818 (0.681, 0.956)	0.365	1.000	0.667
		0.15	0.811 (0.671, 0.950)	0.349	1.000	0.625
		0.05	0.814 (0.675, 0.954)	0.351	1.000	0.667
	Test	0.10	0.690 (0.579, 0.801)	0.291	0.861	0.435
		0.15	0.685 (0.574, 0.796)	0.276	0.889	0.419
		0.05	0.696 (0.586, 0.807)	0.304	0.861	0.435
R5mm + Clinical	Validation	0.10	0.826 (0.689, 0.962)	0.331	1.000	0.625
		0.15	**0.826 (0.689, 0.962)**	**0.396**	**0.909**	**0.750**
		0.05	0.822 (0.685, 0.959)	0.343	1.000	0.667
	Test	0.10	0.690 (0.580, 0.800)	0.251	0.889	0.371
		0.15	0.681 (0.570, 0.792)	0.264	0.889	0.387
		0.05	0.695 (0.585, 0.806)	0.274	0.861	0.403
Tumor + Clinical	Validation	0.10	0.811 (0.670, 0.951)	0.302	1.000	0.583
		0.15	0.803 (0.660, 0.946)	0.285	1.000	0.583
		0.05	0.803 (0.660, 0.946)	0.327	1.000	0.625
	Test	0.10	0.695 (0.584, 0.805)	0.321	0.833	0.419
		0.15	0.699 (0.589, 0.809)	0.306	0.889	0.387
		0.05	**0.700 (0.590, 0.811)**	**0.305**	**0.861**	**0.419**
Tumor + R3mm	Validation	0.05	0.633 (0.414, 0.851)	0.427	0.545	0.833
		0.25	0.621 (0.402, 0.840)	0.162	0.818	0.417
		0.35	0.629 (0.415, 0.842)	0.174	0.818	0.458
	Test	0.05	0.488 (0.370, 0.607)	0.010	1.000	0.016
		0.25	0.504 (0.384, 0.624)	0.073	0.917	0.145
		0.35	0.517 (0.396, 0.637)	0.039	0.944	0.113
Tumor + R5mm	Validation	0.40	0.674 (0.492, 0.856)	0.066	1.000	0.375
		0.45	0.670 (0.485, 0.856)	0.060	1.000	0.375
		0.50	0.659 (0.477, 0.841)	0.055	1.000	0.375
	Test	0.40	0.531 (0.417, 0.645)	0.015	1.000	0.113
		0.45	0.530 (0.416, 0.644)	0.014	1.000	0.113
		0.50	0.528 (0.413, 0.642)	0.176	0.806	0.435

The bold characters represented the AUCs of the optimal models in the validation and test sets.

R5mm + Clinical, model based on radiomics features of the peritumoral 5 mm ring of breast tumor combined with clinical, ABVS, and serology features of breast tumor; R3mm + Clinical, model based on radiomics features of the peritumoral 3 mm ring of breast tumor combined with clinical, ABVS and serology features of breast tumor; Tumor + Clinical, model based on radiomics features of the tumor combined with clinical, ABVS and serology features of breast tumor; Tumor + R5mm, model based on radiomics features of the tumor and those of peritumoral 5 mm ring of breast tumor; Tumor + R3mm, model based on radiomics features of the tumor and those of peritumoral 3 mm ring of breast tumor; AUC, area under the curve; CI, confidence interval.

**Figure 6:**
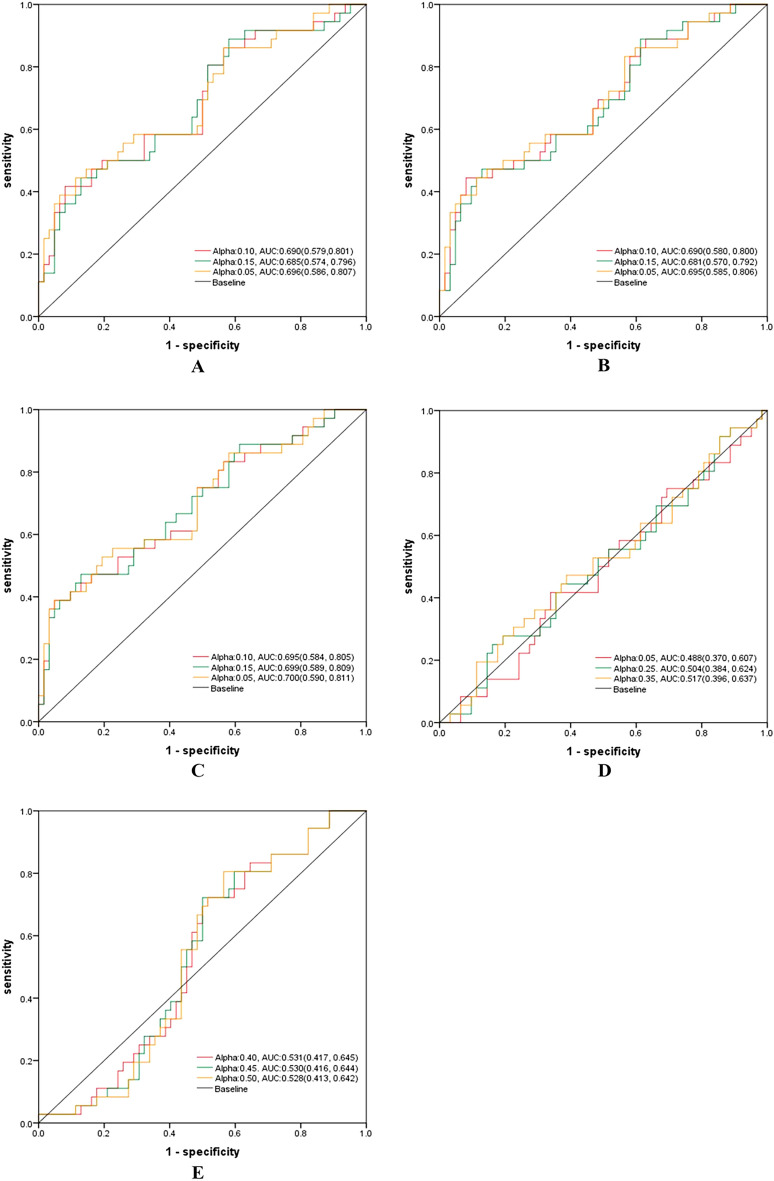
ROCs of weighted combination models based on different alphas in the test set. (**A**) ROC of the R3mm model combined with the Clinical model in the test set. (**B**) ROC of the R5mm model combined with the Clinical model in the test set. (**C**) ROC of the Tumor model combined with the Clinical model in the test set. (**D**) ROC of the Tumor model combined with R3mm model in the test set. (**E**) ROC of the Tumor model combined with R5mm model in the test set. Notes: Alpha, weighting coefficient. AUC, the area under the curve.

In the test set, the Tumor + Clinical model owned the highest AUC, 0.700 (95% CI 0.590–811), the sensitivity of 86.1%, and specificity of 41.9%, which was higher than those of the R3mm + Clinical model, R5mm + Clinical model, Tumor + R5mm model and Tumor + R3mm model (Fig. 6 and Table 3).

In the validation and test set, the AUCs of the weighted combination models T + Clinical, T + R5mm, and T + R3mm were better than those of the corresponding single data source model.

### Comparison of the feature combination models

The AUC, sensitivity, and specificity of the feature combination models in the validation and test sets were shown in S3 Table. In the validation set, the Tumor + Clinical model had the highest AUC, 0.739 (95% CI 0.556–0.921), with a sensitivity of 81.8% and specificity of 66.7%. In the test set, the Tumor + R3mm model owned the best AUC, 0.668 (95% CI 0.555, 0.782), with a sensitivity of 61.1% and specificity of 71.0%.

### Comparison among the single data source model, the feature combination models, and the weighted combination model

Overall, the AUCs of the weighted combination model were higher than most of the corresponding feature combination models and single data source models in both the validation set and the test set. In the validation set, the AUC of the optimal weighted combination model was superior to the optimal feature combination model (0.826 vs. 0.739, P = 0.038), and the optimal single data source model (0.826 vs.0.803, P = 0.446); in the test set, the AUC of the optimal weighted combination model was higher than the optimal feature combination model (0.700 vs. 0.668, P = 0.054), and the optimal single data source model (0.700 vs. 0.695, p = 0.501).

### Features analysis of models

**Figure 7:**
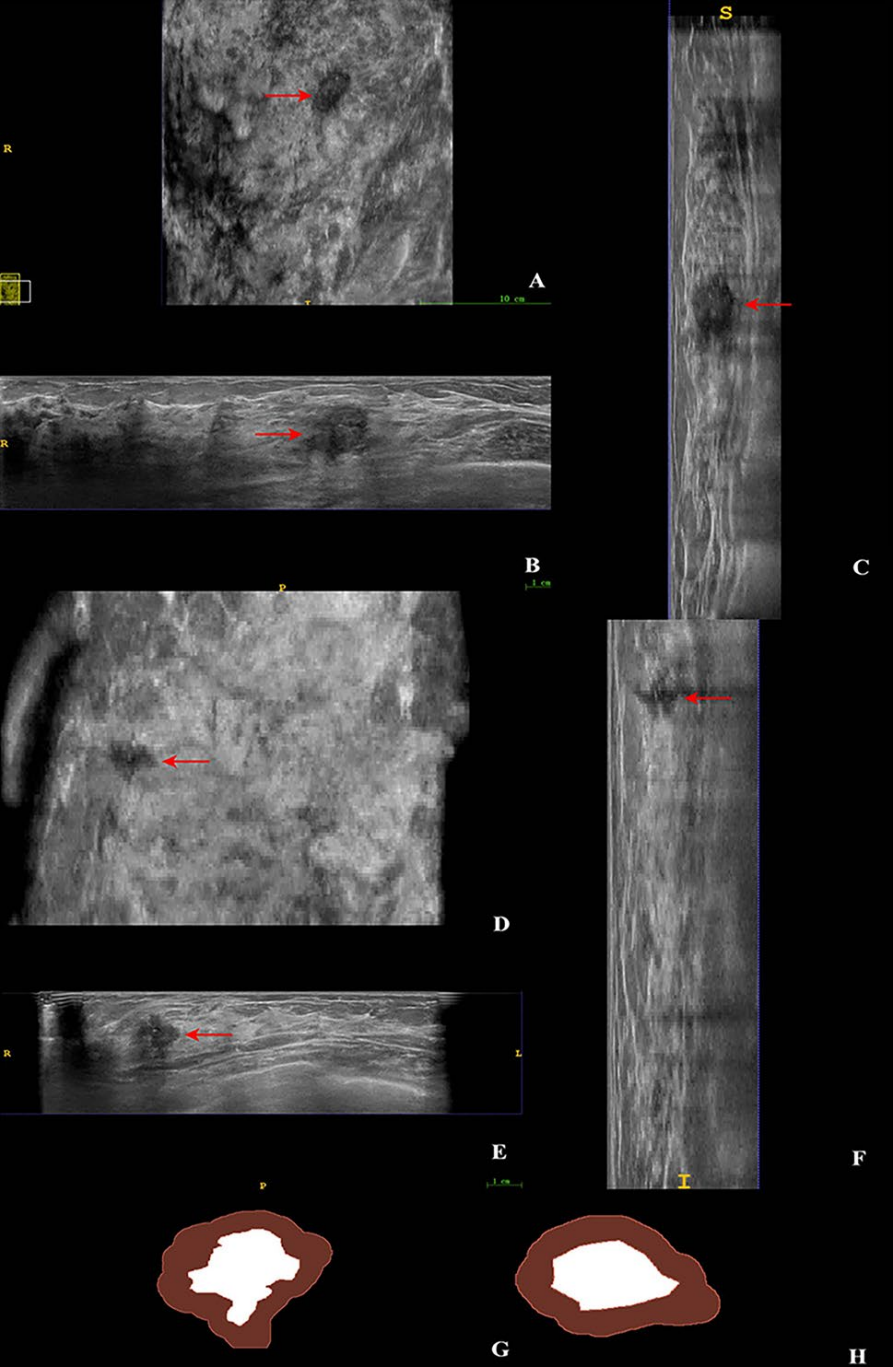
ABVS images of HER2-positive breast tumor and ROIs of the breast tumor region and 5 mm peritumoral region. Notes: Internal microcalcifications in HER2-positive breast cancer in ABVS images (**A** coronal plane, **B** axial plane, **C** sagittal plane; Red arrow identified breast tumor); HER2-positive Breast Cancer Aspect Ratio > 1 in ABVS images (**D** coronal plane, **E** axial plane, **F** sagittal plane; Red arrow identified breast tumor); ROIs of HER2-positive breast tumor region (white area) and 5 mm peritumoral region (red area) were both irregular in shape (**G**); ROIs of HER2-negavive breast tumor region (white area) and 5 mm peritumoral region(red area) were both relative regular in shape (**H**).

R5mm + Clinical and Tumor + Clinical were the optimal radiomics models in the validation and test sets, respectively. Important features for constructing R5mm, Tumor, and Clinical models were shown in S4 Table. For the Clinical model, microcalcifications and aspect ratios were important features in predicting HER2 status, and HER2-positive breast cancers were more likely to show intralesional microcalcifications and growth perpendicular to the skin, as shown in Fig. 7A–C. For the R5mm and Tumor models, Shape_Spherical Disproportion, Shape_Compactness 1, Shape_Compactness 2, and Shape_Elongation features representing tumor shape were key features in predicting HER2 status, and HER2-positive breast cancers tended to be more irregular shapes in the tumor, peritumoral areas (Fig. 7D–F), and ROIs (Fig. 7G–H).

## Discussion

It is an indisputable fact that HER2 is a key therapeutic target for breast cancer. HER2 status is clinically crucial for delaying HER2-positive breast cancer progression, reducing the risk of recurrence^19,20^, improving treatment outcomes^21^, and survival^19,21^. We explored the efficacy of ABVS imaging in predicting HER2 status in breast cancer. Research related to the current study focused on the internal features of breast cancers, ignoring peritumoral information^9,10^. We concluded that peritumoral tissue could provide as much important information as the tumor itself. In the current research, R5mm + Clinical was the optimal weighted combination model in the validation set, and R3mm + Clinical was the weighted combination model second only to Tumor + Clinical in the test set. This was in part consistent with the previous view that peritumoral information has diagnostic and predictive value for molecular typing of breast cancer^22–24^.

The study confirmed that the model weighted combination method had more advantages than the feature combination method in optimizing the model, and we speculated that the model weighted combination method could preserve and optimize the vital features of the single data source model^25^. Some studies that predicted HER2 status in breast cancer focused only on image features and did not include relevant clinical data as predictors^15^, which might lead to the low performance of the model (AUC:0.650). In this study, clinical models, as the optimal single data source model, contributed significantly to the predictive performance of weighted combined models. The reason might be that the ABVS ultrasound features included in the Clinical model could largely reflect tumor heterogeneity^26^, which was also identical to the findings of Zheng et al^27^. ABVS, as a three-dimensional breast ultrasound, could provide more comprehensive breast tumor information than conventional ultrasound, which was presumed to be the reason why the AUC of the ABVS-based radiomics model in the current study was higher than that of the conventional ultrasound-based radiomics model (AUC: 0.826 vs. 0.740)^16^.

We compared the predictive performance of different classifiers when constructing models of intratumoral, peritumoral, and clinical features to select the optimal classifier, optimizing the predictive performance of the single data source model to some extent. Because of the important significance of HER2 positivity for breast cancer diagnosis and treatment, we optimized the cutoff value of ROC to ensure the high sensitivity of the model.

In the current study, we derived important features for constructing the Tumor, R5mm, and Clinical models. In terms of the Tumor model, leukocytes, basic granulocytes, microcalcifications, aspect ratio, and postoperative axillary lymph node metastasis status were crucial features that predict HER2 positivity. This coincided with previous studies demonstrating that basic granulocytes, monocytes, lymphocytes, and microcalcifications were predictors of HER2-positive breast cancer^28–30^. Aspect ratio ≥ 1 and axillary lymph node metastasis were the manifestations of invasive growth and metastasis of breast cancer, which owned a certain predictive value for HER2-positive breast cancer with higher invasiveness^4^. For R5mm and Clinical models, Shape_SphericalDisproportion, Shape_Compactness 1, Shape_Compactness 2, and Shape_Elongation features representing tumor shape could reflect tumor aggressiveness. In addition, GLSZM, GLCM, and GLRLM, as grayscale features, might reflect the heterogeneity and complexity of tumors^31^.

Given the performance of the single data source model was unsatisfactory in the validation set, we utilized the method of model weighted to construct a combined model. At the same time, to obtain the optimal weighting coefficient (alpha), we drew an alpha-AUC scatter plot to accurately and intuitively acquire the tendency of the AUC of the weighted combined model to change with alpha. The above method fully optimized the prediction performance of the weighted combination model.

In the present research, the performance of the weighted combination model was significantly superior to that of the feature combination model, but it was not much different from the single data source model. We will explore a more effective combination mode to optimize the combination model in the future.

Our study had the following strengths: Firstly, the multicenter test set was acquired to assess the clinical generalizability of the model. Secondly, combining ABVS radiomics features with relevant clinical and serological features greatly enhanced the predictive performance of the model. Thirdly, the optimal combination model was constructed by the model weighted combination method based on the idea of model ensemble. Forth, with 3D ultrasound data, we're able to provide more comprehensive tumor information for model construction.

The performance of the proposed model may be influenced by the clarity of the ABVS images, the precision of the ROIs, the accuracy of the ABVS ultrasound and the serological features. While the present study showed promising results for predicting HER2 status in breast cancer. However, it also had several limitations: First of all, further research on breast cancer molecular subtypes and assessment of response to neoadjuvant chemotherapy are still needed. In addition, the model should be applied to other ultrasound modalities such as contrast-enhanced ultrasound and ultrasound elasticity^32,33^. Furthermore, future studies should also expand the number of cases to include other types of breast cancer, verifying the generalizability of the modely^34,35^. Last but not least, how to apply the research results to clinical practice was the direction that we should strive for in the future.

In summary, the weighted combination model integrating ABVS imaging features, and clinical and serological features could better predict HER2 status in breast cancer patients than the feature combination model and had certain clinical generalizations. The current study provided a simple, non-invasive, and preoperative method for HER2 status prediction, guiding the individualized clinical decision-making for breast cancer patients.

### Supplementary Information

Supplementary Information.

## Data Availability

The datasets generated and/or analysed during the current study are not publicly available due to protect the privacy of the patients, but are available from the corresponding author on reasonable request.
