# Supplementary material for: Intra- and peritumoral radiomics features based on multicenter automatic breast volume scanner for noninvasive and preoperative prediction of HER2 status in breast cancer: a model ensemble research
Source: Sci Rep. 2024 Feb 29;14:5020. doi: 10.1038/s41598-024-55838-4 (PMC10904744; doi:10.1038/s41598-024-55838-4)
Supplement: Supplementary file 1 — Supplementary Information. [file 41598_2024_55838_MOESM1_ESM.docx]

**S1 Table. ABVS ultrasound features of patients with breast cancer.**

| **Features** | **Training set (N=139)** | | ***P* value** | **Validation set (N=35)** | | ***P* value** |
| --- | --- | --- | --- | --- | --- | --- |
|  | **HER2+（n=45）** | **HER2- (n=94)** |  | **HER2+**  **(n=11)** | **HER2-**  **(n=24)** |  |
| **Ultrasound-reported ALN status** |  |  | 0.984 ^b^ |  |  | 0.189 ^b^ |
| **Positive** | 13 | 27 |  | 1 | 7 |  |
| **Negative** | 32 | 67 |  | 10 | 17 |  |
| **Lesion maximum diameter (mm)** | 16.00(10.00,20.50) | 18.50(12.50,22.00) | 0.070^c^ | 13.00(4.00,20.00) | 20.0(15.50,32.63) | 0.036^c^ |
| **Boundary** |  | | 0.021 ^b*^ |  | | 0.282 ^b^ |
| **Smooth** | 2 | 18 |  | 3 | 3 |  |
| **No smooth** | 43 | 76 |  | 8 | 21 |  |
| **Shape** |  | | 0.023 ^b*^ |  | | 0.031^b*^ |
| **Regular** | 5 | 2 |  | 2 | 0 |  |
| **Irregular** | 40 | 92 |  | 9 | 24 |  |
| **Aspect ratio** |  | | 0.031 ^b*^ |  | | 0.028^b*^ |
| **＜1** | 30 | 78 |  | 4 | 18 |  |
| **≥1** | 15 | 16 |  | 7 | 6 |  |
| **Hale** |  | | 0.014^b*^ |  | | 0.941 ^b^ |
| **Yes** | 27 | 75 |  | 10 | 22 |  |
| **No** | 18 | 19 |  | 1 | 2 |  |
| **Internal components** |  | | 0.324 ^b^ |  | | NA |
| **Solid** | 45 | 92 |  | 11 | 24 |  |
| **Others** | 0 | 2 |  | 0 | 0 |  |
| **Echo** |  | | 0.547 ^b^ |  | | NA |
| **Low echo** | 44 | 90 |  | 11 | 24 |  |
| **Others** | 1 | 4 |  | 0 | 0 |  |
| **Microcalcification** |  | | 0.039 ^b^ |  | | 0.677 ^b^ |
| **Yes** | 29 | 43 |  | 7 | 15 |  |
| **No** | 16 | 51 |  | 3 | 9 |  |
| **Convergence sign** |  | | 0.197^b^ |  | | 0.560^b^ |
| **Yes** | 32 | 76 |  | 10 | 23 |  |
| **No** | 13 | 18 |  | 1 | 1 |  |

^a^ T-test. ^b^ Chi-square test. ^c^ Mann Whitney U test. *P value < 0.05.

ABVS, automatic breast volume scanner; ALN, axillary lymph node；ALNM, axillary lymph node metastasis; NA, not available

**S2 Table.** **Serological indicators of patients with breast cancer.**

| **Indicators** | **Training set (N=139)** | | ***P* value** | **Validation set (N=35)** | | ***P* value** |
| --- | --- | --- | --- | --- | --- | --- |
|  | **HER2+（n=45）** | **HER2-**  **(n=94)** |  | **HER2+**  **(n=11)** | **HER2-**  **(n=24)** |  |
| **Erythrocyte** | 4.27±0.65 | 4.38±0.50 | 0.264 ^a^ | 4.60±0.41 | 4.39±0.57 | 0.276 ^a^ |
| **Hemoglobin** | 128.00(124.00,139.50) | 132.00(123.00, 144.25) | 0.240 ^b^ | 142.00(135.00,163.00) | 130.50(126.50,143.75) | 0.010 ^b*^ |
| **Erythrocyte pressure** | 39.20(36.90,42.00) | 39.35(37.30,42.53) | 0.311 ^b^ | 43.30(41.10,49.60) | 39.45(38.40,43.25) | 0.019 ^b*^ |
| **Mean erythrocyte volume** | 90.00(86.20,92.75) | 91.10(87.80,94.38) | 0.015 ^b*^ | 95.80(94.30,97.10) | 91.60(88.83,95.48) | 0.022 ^b*^ |
| **Mean hemoglobin content** | 29.70(27.70,31.20) | 30.60(29.35,31.60) | 0.027 ^b*^ | 31.50(31.50,32.90) | 30.50(29.80,32.20) | 0.042 ^b*^ |
| **Mean hemoglobin concentration** | 327.00(322.00,336.00) | 330.00(321.00,335.50) | 0.455 ^b^ | 329.00(328.00,339.00) | 331.50(324.50,334.75) | 0.606 ^b^ |
| **Erythrocyte distribution width（SD）** | 46.20(42.20,47.70) | 45.1(42.85,50.23) | 0.955 ^b^ | 46.40(45.0,47.50) | 71.90(43.93,50.50) | 0.569 ^b^ |
| **Erythrocyte distribution width（CV）** | 14.40(13.30,15.55) | 13.50(13.10,15,13) | 0.227 ^b^ | 13.30(13.10,14.70) | 14.05(13.30,15.05) | 0.123 ^b^ |
| **Leukocyte** | 4.57(3.83,5.70) | 5.43(4.30,6.84) | 0.008 ^b*^ | 4.81(4,61,5.46) | 4.78(3.77,6.07) | 0.859^b^ |
| **Lymphocyte Percentage** | 28.50(23.70,34.45) | 25.90(20.18,30.85) | 0.213 ^b^ | 25.10(23.70,37.10) | 27.850(25.55,33.48) | 0.414 ^b^ |
| **Monocyte percentage** | 7.40(5.20,9.35) | 6.30(4.40,7.80) | 0.062 ^b^ | 6.10(5.10,8.10) | 6.20(4.73,8.18) | 0.569 ^b^ |
| **Neutrophil percentage** | 65.00(57.85,72.55) | 61.00(56.70,67.60) | 0.094 ^b^ | 65.80(53.50,68.70) | 60.55(56.33,68.63) | ^1.000 b^ |
| **Eosinophil percentage** | 1.00(0.40，1.80) | 0.95 (0.50，1.80) | 0.579 ^b^ | 0.90(0.60，1.10) | 1.10(0.53，1.88) | 0.557 ^b^ |
| **Basophil percentage** | 0.30 (0.20，0.75) | 0.30(0.20，0.60) | 0.991 ^b^ | 0.20(0.20，0.20) | 0.30(0.20，0.68) | 0.017 ^b*^ |
| **Lymphocyte absolute value** | 1.40(1.06,1.79) | 1.41(1.10,1.80) | 0.340 ^b^ | 1.37(1.10,2.00) | 1.39(1.11,1.59) | 0.943 ^b^ |
| **Monocyte absolute value** | 0.31(0.25，0.43) | 0.33(0.26，0.46) | 0.746 ^b^ | 0.36(0.28，0.40) | 0.32(0.25，0.41) | 0.844 ^b^ |
| **Neutrophil absolute value** | 2.95(2.16,3.45) | 3.72(2.49,4.85) | 0.010 ^b*^ | 3.20(2.27，3.75) | 2.90(2.36，3.79) | 0.972 ^b^ |
| **Eosinophil absolute value** | 0.04(0.02，0.09) | 0.06(0.02，0.09) | 0.227 ^b^ | 0.05(0.02，0.05) | 0.05(0.02，0.10) | 0.517 ^b^ |
| **Basophil absolute value** | 0.01(0.01，0.03) | 0.02(0.01，0.03) | 0.615 ^b^ | 0.01(0.01，0.01) | 0.02(0.00，0.03) | 0.264 ^b^ |
| **Platelet** | 231.40±84.50 | 238.78±83.24 | 0.953 ^a^ | 228.27±43.63 | 216.25±74.78 | 0.494 ^a^ |
| **Platelet ratio** | 0.23(0.20,028) | 0.24(0.20,0.29) | 0.464 ^b^ | 0.23±0.04 | 0.24±0.07 | 0.032 ^a*^ |
| **Mean platelet volume** | 10.74±1.40 | 10.56±1.17 | 0.204 ^a^ | 10.39±1.00 | 11.47±1.24 | 0.756 ^a^ |
| **Platelet distribution width** | 15.50(11.90,16.10) | 14.11(12.25,16.15) | 0.803 ^b^ | 13.32±1.71 | 14.99±3.15 | 0.047 ^a*^ |
| **Large platelet ratio** | 31.56±10.35 | 29.66±9.23 | 0.405 ^a^ | 33.90(17.70,34.70) | 36.60(30.93,41.88) | 0.017 ^b*^ |
| **CA153** | 13.97(8.65，18.30) | 14.40(9.27，20.35) | 0.451 ^b^ | 17.90(17.90，21.10) | 14.80(9.18，24.70) | 0.455 ^b^ |
| **CA125** | 12.80(8.74,17.90) | 14.35(9.95,18.58) | 0.069 ^b^ | 13.70(10.90,17.60) | 17.85(10.13,28.15) | 0.477 ^b^ |
| **CEA** | 1.40(1.20，2.25) | 1.85 (1.20，2.70) | 0.221 ^b^ | 1.70(1.50，2.30) | 1.80 (1.15，2.28) | 0.499 ^b^ |
| **Total bilirubin** | 11.60（9.90,13.60） | 12.90（8.70,15.43） | 0.982 ^b^ | 11.20(9.30,21.30) | 11.50(8.53,15.08) | 0.394 ^b^ |
| **Direct bilirubin** | 1.90(1.60,2.75) | 2.35(1.68,3,13) | 0.210 ^b^ | 2.00(1.60,3.60) | 2.20(1.63,2.98) | 0.776 ^b^ |
| **Indirect bilirubin** | 9.80(8.05,11.45) | 8.95(6.80,11.58) | 0.158 ^b^ | 9.20(7.80,17.70) | 9.45(6.65,12,10) | 0.413 ^b^ |

^a^ T-test; ^b^ Mann Whitney U test; *P value < 0.05.

SD, standard deviation; CV, coefficient of variation; CA, cancer antigen; CEA, cancer embryo antigen

**S3 Table.** **Predictive performance for HER2 state of feature combination models based on a variety of classifiers, in the training, the validation, and the test set.**

| **Feature combination models** | **Sets** | **Classifiers** | **AUC（95%CI）** | **Cutoff** | **Sensitivity** | **Specificity** |
| --- | --- | --- | --- | --- | --- | --- |
| **R3mm+Clinical** | Validation | GBC | 0.682(0.481,0.883) | 0.376 | 0.636 | 0.167 |
|  |  | LGBM | 0.580(0.366.0.793) | 0.076 | 0.273 | 1.000 |
|  |  | RFC | 0.534(0.325,0.743) | 0.575 | 0.364 | 0.792 |
|  | Test | GBC | 0.540(0.420,0.661) | 0.516 | 0.222 | 0.708 |
|  |  | LGBM | 0.453(0.332,0.574) | 0.376 | 0.194 | 0.871 |
|  |  | RFC | 0.491(0.369,0.614) | 0.409 | 0.278 | 0.806 |
| **R5mm+Clinical** | Validation | GBC | 0.652(0.444,0.859) | 0.286 | 0.636 | 0.708 |
|  |  | ETC | 0.479(0.262,0.696) | 0.474 | 0.455 | 0.708 |
|  |  | RFC | 0.583(0.364,0.802) | 0.499 | 0.455 | 0.792 |
|  | Test | GBC | 0.460(0.341,0.580) | 0.034 | 0.639 | 0.419 |
|  |  | ETC | 0.487(0.366,0.608) | 0.530 | 1.000 | 1.000 |
|  |  | RFC | 0.523(0.404,0.642) | 0.353 | 0.556 | 0.548 |
| **Tumor+Clinical** | Validation | GBC | **0.739(0.556,0.921)** | 0.248 | 0.818 | 0.667 |
|  |  | LGBM | 0.542(0.307,0.776) | 0.492 | 0.364 | 0.917 |
|  |  | RFC | 0.610(0.411,0.808) | 0.106 | 0.727 | 0.583 |
|  | Test | GBC | 0.492(0.370,0.615) | 0.744 | 0.139 | 0.968 |
|  |  | LGBM | 0.567(0.447,0.686) | 0.193 | 0.611 | 0.581 |
|  |  | RFC | 0.555(0.437,0.672) | 0.017 | 0.722 | 0.403 |
| **Tumor+R3mm** | Validation | GBC | 0.542(0.279,0.804) | 0.802 | 0.364 | 1.000 |
|  |  | LGBM | 0.394(0.167,0.621) | 0.547 | 0.364 | 0.750 |
|  |  | RFC | 0.424(0.205,0.644) | 0.984 | 0.182 | 0.917 |
|  | Test | GBC | **0.668(0.555,0.782)** | 0.095 | 0.611 | 0.710 |
|  |  | LGBM | 0.547(0.426,0.668) | 0.389 | 0.583 | 0.581 |
|  |  | RFC | 0.623(0.501,0.744) | 0.850 | 0.583 | 0.758 |
| **Tumor+R5mm** | Validation | GBC | 0.458(0.252,0.665) | 0.291 | 0.545 | 0.625 |
|  |  | ABC | 0.360(0.172,0.548) | 0.421 | 1.000 | 0.042 |
|  |  | ETC | 0.417(0.201,0.632) | 0.596 | 0.182 | 0.875 |
|  | Test | GBC | 0.578(0.460,0.696) | 0.060 | 0.806 | 0.403 |
|  |  | ABC | 0.456(0.334,0.578) | 0.502 | 0.139 | 0.935 |
|  |  | ETC | 0.581(0.464,0.697) | 0.266 | 0.667 | 0.516 |

The bold characters represented the AUCs of the optimal models in the validation and test sets. R5mm+Clinical, radiomics features of the peritumoral 5mm ring of breast tumor combined with clinical, ABVS and serology features of breast tumor; R3mm+Clinical, radiomics features of the peritumoral 3mm ring of breast tumor combined with clinical, ABVS and serology features of breast tumor; Tumor+Clinical, radiomics features of the tumor combined with clinical, ABVS and serology features of breast tumor; Tumor+R5mm, radiomics features of the tumor combined with those of peritumoral 5mm ring of breast tumor; Tumor+R3mm, radiomics features of the tumor combined with those of peritumoral 3mm ring of breast tumor; AUC, area under the curve; CI, confidence interval

**S4 Table. The first 10 features for constructing the Clinical, Tumor and R5mm models**

| Models | Features name |
| --- | --- |
| Clinical | Eosinophils cells absolute value |
|  | Eosinophils percentage |
|  | Basophil percentage |
|  | Leukocyte |
|  | Microcalcifications |
|  | Total bilirubin |
|  | Erythrocyte |
|  | Status of axillary lymph node metastasis |
|  | Aspect Ratio |
|  | Basophil cells absolute value |
| Tumor | Log_GLRLM_LongRunEmphasis |
|  | Wavelet_GLSZM_SmallAreaEmphasis |
|  | Log_Firstorder_Maximum |
|  | Log_GLSZM_GrayLevelNonUniformityNormalized |
|  | Wavelet_GLSZM_GrayLevelVariance |
|  | Wavelet_GLCM_ClusterShade |
|  | Original_shape_SphericalDisproportion |
|  | Original_shape_Compactness2 |
|  | Wavelet_GLSZM_LowGrayLevelZoneEmphasis |
|  | Original_shape_Compactness1 |
| R5mm | Wavelet_GLSZM_LargeAreaHighGrayLevelEmphasis |
|  | Log_GLCM_Contrast |
|  | Log_NGTDM_Strength |
|  | Wavelet_GLSZM_GrayLevelNonUniformityNormalized |
|  | Wavelet_GLSZM_LargeAreaHighGrayLevelEmphasis |
|  | Log_GLCM_IMC2 |
|  | Log_NGTDM_Strength |
|  | Original_shape_Elongation |
|  | Wavelet_GLCM_IMC2 |
|  | Wavelet_GLSZM_GrayLevelVariance |

Clinical, clinical, ABVS and serology features of breast tumor; R5mm, peritumoral 5mm ring of breast tumor; Tumor, radiomics features of the tumor
